# Different Types of Door-Opening Motions as Contributing Factors to Containment Failures in Hospital Isolation Rooms

**DOI:** 10.1371/journal.pone.0066663

**Published:** 2013-06-24

**Authors:** Julian W. Tang, Andre Nicolle, Jovan Pantelic, Christian A. Klettner, Ruikun Su, Petri Kalliomaki, Pekka Saarinen, Hannu Koskela, Kari Reijula, Panu Mustakallio, David K. W. Cheong, Chandra Sekhar, Kwok Wai Tham

**Affiliations:** 1 Department of Laboratory Medicine, National University Hospital, Singapore; 2 Alberta Provincial Laboratory for Public Health, Edmonton, Alberta, Canada; 3 Department of Medical Microbiology and Immunology, University of Alberta, Edmonton, Alberta, Canada; 4 Department of Building, School of Design and Environment, National University of Singapore, Singapore; 5 Finnish Institute of Occupational Health, Helsinki, Finland; 6 Oy Halton Group Ltd, Vantaa, Finland; University Hospital San Giovanni Battista di Torino, Italy

## Abstract

Hospital isolation rooms are vital for the containment (when under negative pressure) of patients with, or the protection (when under positive pressure) of patients, from airborne infectious agents. Such facilities were essential for the management of highly contagious patients during the 2003 severe acute respiratory syndrome (SARS) outbreaks and the more recent 2009 A/H1N1 influenza pandemic. Many different types of door designs are used in the construction of such isolation rooms, which may be related to the space available and affordability. Using colored food dye as a tracer, the qualitative effects of door-opening motions on the dissemination of potentially contaminated air into and out of a single isolation room were visualized and filmed using Reynolds-number-equivalent, small-scale, water-tank models fitted with programmable door-opening and moving human figure motions. Careful scaling considerations involved in the design and construction of these water-tank models enabled these results to be accurately extrapolated to the full-scale situation. Four simple types of door design were tested: variable speed single and double, sliding and hinged doors, in combination with the moving human figure. The resulting video footage was edited, synchronized and presented in a series of split-screen formats. From these experiments, it is clear that double-hinged doors pose the greatest risk of leakage into or out of the room, followed by (in order of decreasing risk) single-hinged, double-sliding and single-sliding doors. The relative effect of the moving human figure on spreading any potential contamination was greatest with the sliding doors, as the bulk airflows induced were large relative to those resulting from these door-opening motions. However, with the hinged doors, the airflows induced by these door-opening motions were significantly greater. Further experiments involving a simulated ventilated environment are required, but from these findings alone, it appears that sliding-doors are far more effective for hospital isolation room containment.

## Introduction

Isolation rooms to contain infectious patients or to protect vulnerable (e.g. immunocompromised) patients from infection are an important facility to protect patients and staff against the risk of infection by airborne pathogens [1], [2]. Recommendations for their use features in many guidelines related to the control of airborne pathogens, especially for tuberculosis [3]–[6]. In the aftermath of the severe acute respiratory syndrome outbreaks of 2003, the demand for such rooms increased, dramatically [7]–[10]. Many of these were eventually utilized in the management of patients infected with the 2009 pandemic influenza A/H1N1 virus [11]–[14].

Although there have been many studies evaluating the performance of such rooms with regard to the maintenance of the pressure differential across the doors when closed [15]–[18], there have been relatively fewer studies assessing how door-opening motions and healthcare worker passage through the door can affect the performance of such rooms [19]–[22]. Yet, at least one analytical case report has demonstrated that containment failure may result from simply opening isolation room doors [23].

This study is part of a longer-term project that aims to demonstrate the effects of door-opening motions using a variety of doors, with and without the passage of a human figure, on the movement of potentially contaminated air into and out of an isolation room, using both a small-scale, Reynolds-number-equivalent model in water, and a full-scale model in air. In this study, baseline measurements were made using colored food dye visualization in still water (i.e. to simulate still air) for each of the moving figure-door systems, with no simulated ventilation system imposed.

## Methods

It was decided to conduct the experiments in water, at Reynolds number equivalent lengths and velocities, such that the results obtained in water could be directly extrapolated to the full-scale situation in air [20]. Water was chosen because it was easier to visualize flows, qualitatively, using coloured dye, or, more quantitatively, using neutrally buoyant, suspended, reflective particles for particle image velocimetry (PIV).

### Water-tank Models

**Figure 1:**
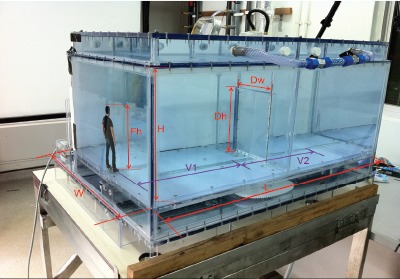
Dimensions of hinged−/sliding-door experimental scale-model water-tanks (both identical). L – tank length (0.81 m); W – tank width (0.47 m); H – tank height (0.30 m); Dh – door height (0.205 m); Dw – door gap width (0.11 m single, 0.19 m double); Fh – figure height (0.175 m); V1, V2 - figure velocities through Stages 1, 2 respectively.

**Figure 2:**
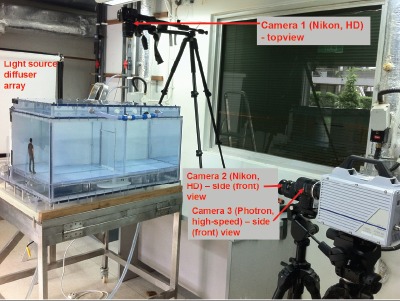
Photograph of camera and light-source layout. These images were taken at the stage just before the addition of the colored food dye to one of the chambers in the experimental water-tank.

Two simplified, one-tenth scale (1∶10) models were designed and constructed, as shown in Figures 1 and 2 (CYS Engineering & Trading, Singapore). All the dimensions of the one-tenth scale models were taken from full-scale models that were being constructed at the same time by collaborators at the Finnish Institute of Occupational Health (FIOH) and their collaborators Oy Halton Group (Finland). One of the larger aims of this study involving the parallel construction of these two models was to test and validate more fundamental scaling assumptions about flow dynamics, using flow visualization methods as well as computational fluid dynamical (CFD) modeling.

One tank was designed to accommodate single- and double-sliding doors, the other single- and double-hinged doors, as the door-opening mechanisms were significantly different for these two types (i.e. sliding versus hinged) of doors. Each of the tanks had interchangeable door modules so that the single- and double- version of the doors could be swapped as required for the experiments. Otherwise the tanks were of virtually identical dimensions. The same scale model of a human male figure was used in each tank to maintain similarity. The figure moved on a sliding track that was built into the floor of each tank, and which could be programmed to move at realistic human walking speeds. This track was regularly greased to ensure a smooth sliding action without juddering. The movement controller chip (Arduino Mega 1280, ATmega1280 (Silicon Core), Arduino, Chiasso, Switzerland) for each of the tanks was manually programmed to allow the figure and doors to move at speeds similar to those observed in real isolation facilities.

### Experimental Procedure

Once each of the figure-door movement systems in each tank was reset, the edges around the door module (including the door and surrounding wall partition) were carefully sealed, manually, with Vaseline® Petroleum Jelly (Unilever, London, UK) to prevent any leakage of the food dye across the door partition prior to the running of the experiment. Once the seal was in place, the tank was slowly filled with water from the bottom up via a supply pipe entering the floor of the tank. This facilitated the removal of air bubbles from the tank, as it filled, which would otherwise obstruct clear views of the tank from the top that were required for filming. The tank was also placed on a custom-built table which allows a tilt of up to 45° when the tank was full, to allow any air bubbles to escape through small drainage air-holes that were drilled into the roof along its edge. Any remaining bubbles were manually suctioned using a 30 ml or 50 ml syringe and small-bore catheter that was passed through these air-holes, as required. Once the bubbles had been removed and the tank leveled again, 50 mls of blue food dye (True Blue, Star Brand, Kuala Lumpur, Malaysia) was injected into one side of the tank, as required by the experimental protocol, i.e. into the side containing the human figure (the ‘from dye’ protocol), or into the side without the figure (the ‘to dye’ protocol). The syringe catheter was used to mix the food dye carefully within the chamber to ensure a relatively uniform dye distribution.

The tank was back-lit using a lighting rig that consisted of a bank of 18 spotlights (240 V, 120 W, Par 38 Spot 12°, Philips, Amsterdam, Holland) arranged in a 3-row by 6-column grid, whose beams were diffused by a series of cloth diffusers and grease-proof paper that was taped to the back of the water-tank model. Three cameras were used to obtain video images: a Nikon D7000 with a 28–105 mm Nikkor AF lens in manual mode (Nikon Inc., Melville, NY), at 24 frames per second (fps), high definition (HD, color, 1920×1280 pixels) from the front; a Nikon 3100 with a 18–55 mm Nikkor AF-S lens in manual mode, at 24 frames per second (fps), high definition (HD, color, 1920×1280 pixels) from the top; a Photron SA1.1 camera (Dynamic Analysis System, Pte Ltd, Singapore), at 500 fps (black and white) with a 28–85 mm Nikkor (manual) lens, also from the front – to allow extreme slow motion playback.

Once the lighting was in place and switched on, and the cameras were in position and recording, the controller was activated and the figure and doors moved according to a pre-set program: the figure would accelerate almost instantly to its designated scaled-down walking speed, slide towards the door. It would then stop just before the door to allow it to open, before passing through the doorway to enter the room. The doors would then close behind it, completing the programmed movement cycle. Note that the figure’s movements were not quite the same for the sliding and hinged doors. A larger clearance in front of the figure was required for the hinged (i.e. when opening towards the figure) than the sliding doors so as to avoid any collision between it and the door.

Several movement combinations were tried with the figure and the various types of doors, including scenarios where the doors opened and closed alone, with no figure movement, as these defined a baseline airflow behavior for the action of these doors in the absence of any human movement. After each experimental run, the tank was drained of water (now coloured with food dye) and cleaned. If necessary, a different door module was inserted and the edges sealed with Vaseline®, before the tank was refilled and the cameras repositioned as required, for the next run.

For this baseline series of experiments, all of them were performed in a still water environment, with no pressure differential simulated across the doorway.

### Scaling Issues and Analysis

The programmed velocities of the door-opening and human figure movements were based on the principles of Reynolds (Re) number equivalence, i.e. that motion in the 1∶10 scale model in water should be equivalent to the same motion at full-size in air, i.e. Re_air_ = U_air_L_air_/ν_air_ = Re_water_ = U_water_L_water_/ν_water_ where U, L and νare the velocity, representative lengths and kinematic viscosities in the two media, respectively. If the length scale in the water-tank model is one-tenth of that in air (i.e. L_air_ = 10L_water_), and the kinematic viscosities of water at 20°C and air at 25°C (the approximate operating temperatures in this tropical climate) are 1.004×10^−6^ m^2^/s and 15.6×10^−6^ m^2^/s, respectively, then the ratio of the velocities U_air_/U_water_ = 1.56/1.004 = 1.55. For the angular velocities of the hinged-doors in air and water (?_air_ and ?_water_, respectively?, their equivalent linear velocities must also scale as U_air_/U_water_ = L_air_?_air_/L_water_?_water_ = 1.55, so that the ratio of the angular velocities are related as ?_air_ = 0.155?_water_, or ?_air_/?_water_ = where L is the width of the door.

Due to the qualitative nature of the food dye tracer used for visualization, the descriptions of its movements are best demonstrated as a series of photographs and videos (available as Videos S1, S2, S3, and S4), with some relative comparisons presented in the Results below. Multiple repeats of each experimental scenario produced very similar results at this qualitative level, which were sufficient to show the relative differences between the performance of the different door and human motion combinations.

## Results

**Figure 3:**
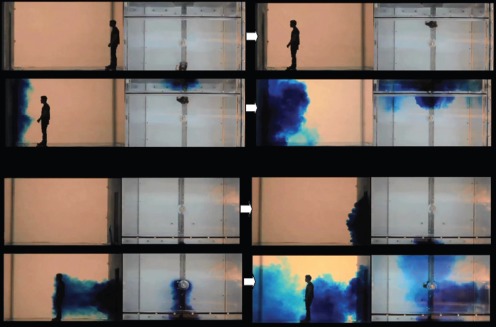
Single-sliding door snapshots (side and top views; left-to-right, top-to-bottom). The series of 4 snapshots with each door-opening, manikin movement scenario were taken with respect to the following events, rather than at specific times: food dye movement due to door-opening motions alone then with any initial manikin movement – manikin interaction and any entrainment food dye – final food dye movements once the manikin had come to rest at its destination position. All movement parameters are shown in **Table 1** for these single-sliding door scenarios. Note that with the sliding doors, the scenarios where the manikin enters or leaves the isolation room are effectively symmetrical (unlike with the hinged-door scenarios). **A.** Manikin moving into/out of the isolation room (seen from outside/inside, respectively), V** = **0.79 in water (1.22 in air) m/s, door-opening gap velocity = 0.24 in water (0.37 in air) m/s. **B.** Manikin moving out of/into the isolation room (seen from outside/inside, respectively), V** = **0.79 in water (1.22 in air) m/s, door-opening gap velocity = 0.24 in water (0.37 in air) m/s.

**Figure 4:**
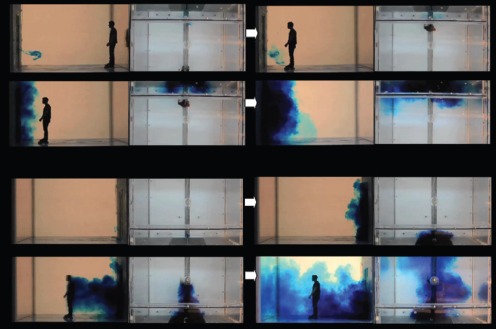
Double-sliding door snapshots (side and top views; left-to-right, top-to-bottom). The series of 4 snapshots with each door-opening, manikin movement scenario were taken with respect to the following events, rather than at specific times: food dye movement due to door-opening motions alone then with any initial manikin movement – manikin interaction and any entrainment food dye – final food dye movements once the manikin had come to rest at its destination position. All movement parameters are shown in **Table 1** for these double-sliding door scenarios. Note that with the sliding doors, the scenarios where the manikin enters or leaves the isolation room are effectively symmetrical (unlike with the hinged-door scenarios). **A.** Manikin moving into/out of the isolation room (seen from outside/inside, respectively), V** = **0.79 in water (1.22 in air) m/s, door-opening gap velocity = 0.42 in water (0.64 in air) m/s. **B.** Manikin moving out of/into the isolation room (seen from outside/inside, respectively), V** = **0.79 in water (1.22 in air) m/s, door-opening gap velocity = 0.42 in water (0.64 in air) m/s.

**Figure 5:**
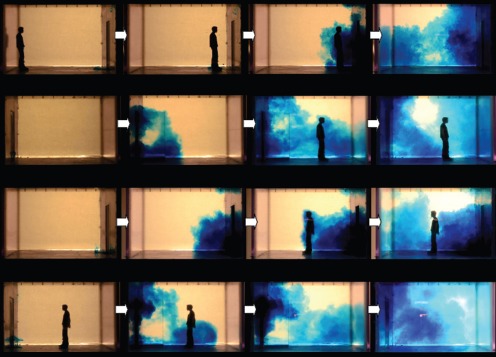
Single-hinged door snapshots (sideviews only; left-to-right). The series of 4 snapshots with each door-opening, manikin movement scenario were taken with respect to the following events, rather than at specific times: food dye movement due to door-opening motions alone then with any initial manikin movement – manikin interaction and any entrainment food dye – final food dye movements once the manikin had come to rest at its destination position. All movement parameters are shown in **Table 2** for these single-hinged door ‘fast’ scenarios. **A.** Manikin moving into the isolation room (seen from outside, V1 = 0.79 in water (1.22 in air) m/s, angular velocity = 184.68 in water (28.63 in air) deg/s.). **B.** Manikin moving into the isolation room (seen from inside), V2 = 0.75 in water (1.17 in air) m/s, angular velocity = 184.68 in water (28.63 in air) deg/s. **C.** Manikin moving out of the isolation room (seen from outside), V** = **0.77 in water (1.19 in air) m/s; angular velocity = 184.68 in water (28.63 in air) deg/s. **D.** Manikin moving out of the isolation room (seen from inside), V** = **0.77 in water (1.19 in air) m/s; angular velocity = 184.68 in water (28.63 in air) deg/s.

**Figure 6:**
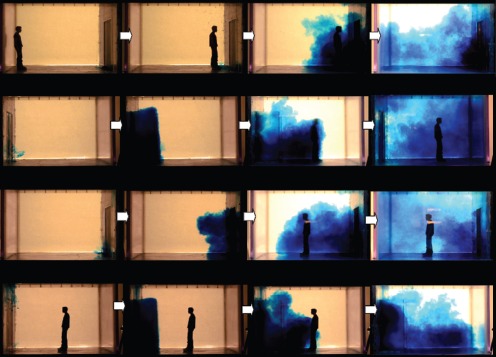
Double-hinged door snapshots (sideviews only; left-to-right). The series of 4 snapshots with each door-opening, manikin movement scenario were taken with respect to the following events, rather than at specific times: food dye movement due to door-opening motions alone then with any initial manikin movement – manikin interaction and any entrainment food dye – final food dye movements once the manikin had come to rest at its destination position. All movement parameters are shown in **Table 2** for these double-hinged door ‘fast’ scenarios. **A.** Manikin moving into the isolation room (seen from outside, V1 = 0.71 in water (1.1 in air) m/s, angular velocity = 163.1 in water (25.3 in air) deg/s.). **B.** Manikin moving into the isolation room (seen from inside), V2 = 0.88 in water (1.36 in air) m/s, angular velocity = 163.1 in water (25.3 in air) deg/s. **C.** Manikin moving out of the isolation room (seen from outside), V** = **0.73 in water (1.14 in air) m/s; angular velocity = 163.1 in water (25.3 in air) deg/s. **D.** Manikin moving out of the isolation room (seen from inside), V** = **0.73 in water (1.14 in air) m/s; angular velocity = 163.1 in water (25.3 in air) deg/s.

Velocities of door-opening and figure movement in each scenario were defined and programmed into the controller chip to be within a realistic parameter range when scaled up to their full-scale motions in an air medium. Realistic walking speeds equivalent to ∼ 1–1.2 m/s in air were chosen for these model parameters, though obviously walking speeds may vary considerably between individuals. Only one sliding door opening-speed was examined (Table 1, Figures 3 and 4, Videos S1–S2), as other settings showed relatively little difference. For the hinged-doors, both slow (Table 1, Videos S3–S4) and fast (Table 2, Figures 5 and 6) angular velocities were investigated. This was done because the effect of these door-opening motions on the movements of the food dye were much more dramatic and it was of interest to capture these flows at these two speeds, with the fast parameters being approximately twice those of the slow parameters. Brief descriptions of each door-opening scenario are also included in Tables 1 and 2.

**Table 1:** Movement parameters of the various door-opening scenarios. (as shown inFigures 3 and 4, and in Videos S1, S2, S3, and S4).

Scenario (video)	Manikin movement velocity in water (air equivalent) (m/s)	Sliding door gap-opening velocity in water (air equivalent) (m/s)	Hinged door-opening angular velocity in water (air equivalent) (degrees/s)	Brief description of flow patterns
Single-sliding door(into room)	0.79 (1.22)	0.24 (0.37)	–	The initial opening of the door produces relatively little leakage across the doorway. The manikin moving through the door causes a large efflux of food dye out of the room as he moves in, and a large movement of dye, entrained into his drag wake, as he moves out
Single-sliding door(out of room)	0.79 (1.22)	0.24 (0.37)	–	
Double-sliding door(into room)	0.79 (1.22)	0.42 (0.64)	–	Similar to the single-sliding door, but with larger volume effluxes. Relatively little leakage occurs across the doorway when the doors open. Significantly more food dye moves across the doorway when the manikin moves into or out of the room, as described above.
Double-sliding door(out of room)	0.79 (1.22)	0.42 (0.64)	–	
Single-hinged door(into room) - slow	V1* = 0.39 (0.61) V2* = 0.34 (0.52)	–	98.2 (15.22)	The hinged-door-opening motion induces a much larger exchange of food dye across the doorway in both directions than the sliding door equivalent. The movement of the manikin through the door adds to this in a delayed manner, but the volume of air moved by the door appears significantly larger. Leading-edge vortices are visible as the door opens and closes, and the food dye contamination of the clean side occurs rapidly.
Single-hinged door(out of room) - slow	0.58 (0.89)	–	98.2 (15.22)	
Double-hinged door(into room) - slow	V1* = 0.35 (0.55) V2* = 0.25 (0.39)	–	86.4 (13.4)	Similar to the single-hinged door, but with larger volume effluxes. The double-hinged-door-opening motions induce significantly more leakage across the doorway than does the manikin moving into or out of the room. Leading-edge vortices are visible as the doors open and close, and the food dye contamination of the clean side occurs extremely rapidly.
Double-hinged door(out of room) - slow	0.53 (0.82)	–	86.4 (13.4)	

* Refer to Figure 1 for the meaning of these V1 and V2 parameters.

**Table 2:** Faster movement parameters for the hinged-door model.

Scenario (video)	Manikin movementvelocity in water(air equivalent) (m/s)	Hinged door-openingangular velocity in water(air equivalent) (degrees/s)	Brief description of flow patterns
Single-hinged door(into room) - fast	V1* = 0.79 (1.22) V2* = 0.75 (1.17)	184.68 (28.63)	Qualitatively similar to the descriptions given in Table 1, but the food dye moves more quickly, as a result of the faster door-opening and manikin movements.
Single-hinged door(out of room) - fast	0.77 (1.19)	184.68 (28.63)	
Double-hinged door(into room) - fast	V1* = 0.71 (1.1) V2* = 0.88 (1.36)	163.07 (25.28)	Qualitatively similar to the descriptions given in Table 1, but the food dye moves more quickly, as a result of the faster door-opening and manikin movements.
Double-hinged door(out of room) - fast	0.73 (1.14)	163.07 (25.28)	

* Refer to Figure 1 for the meaning of these V1 and V2 parameters.

These additional parameter settings were used with relatively similar qualitative outcomes (as shown in Figures 5 and 6).

From a qualitative visual inspection of these images, it can be clearly seen that the single-doors produce less disturbance than the double-doors, and sliding doors produce far less air exchange than hinged-doors, i.e. single-sliding<double-sliding<single-hinged<double-hinged, when the doors are graded in terms of the potential for their door-opening motion to induce bulk air flow movement across the doorways.

For both the single- and double-sliding doors, the movement of the human figure through the door caused a significant additional amount of food dye to be exchanged (both into and out of the room) because the motion of the sliding doors themselves caused very little disturbance to the food dye. In contrast, the relative effect of the manikin movement on the food dye was much smaller when moving through the single- and double-hinged doors because the opening motion of these hinged doors caused significantly more movement of the food dye across the doorway.

## Discussion

There have been relatively few formal, published studies on the effects of door-opening motions on the integrity of containment in hospital isolation rooms, and even fewer where the effects of a healthcare worker moving through the doorway has also been considered. This study aims to fill some of these gaps in our knowledge.

Overall, the results of this qualitative visualization study are not surprising and relatively intuitive, however being able to visualize these relative differences may emphasize the advantages and disadvantages of these different door designs in a more emphatic manner for consideration by hospital managers and administrators, infection control and hospital building design teams.

For general infection control purposes, it is clear that the sliding doors (single or double) offer some obvious advantages over the more conventional hinged-door design in the amount of air exchanged across isolation room doorways each time they are opened. Yet, the majority of hospital isolation rooms still use the more traditional hinged-door design. This may possibly be due to the space requirements and the practicalities of higher installation (sliding doors may be more expensive to make and install) and maintenance costs (there are more moving parts in a sliding- compared to a hinged-door). In addition, where air-tight, ‘non-leaky’ containment facilities are required, it is much easier to ensure an airtight seal around a hinged-door than a sliding-door.

An early study used tracer gas (SF_6_) and measured how its concentration changed when a technician exited the isolation room into an adjoining anteroom and then the outside corridor. Other variables included the time intervals of sampling from the anteroom and the outside corridor, the room size (31.3–49.3 m^3^) and the ventilation rate (15–21 air changes/hour). The results showed that the concentration of the tracer gas decreased dramatically, as measured by a ‘dilution factor’ that was defined by the authors, ranging from 122–211 (5 minutes after gas release) between the isolation room and the anteroom, and 1260–3670 (10 minutes after gas release) between the isolation room and the outside corridor [19]. However, the types of doors used for the isolation and anterooms (i.e. hinged or sliding) were not described, though it was probably a hinged door design. It can be seen from the results presented here that the type of door may dramatically affect the results as hinged door motions can rapidly accelerate any mixing and therefore have an impact on the apparent ‘dilution factor’ that is measured.

Tang et al. [23] described a clinical situation where a severe case of adult chickenpox (i.e. primary varicella zoster virus, VZV, infection) managed in a negative pressure isolation room (with no anteroom) caused an infection of a VZV-susceptible nurse whose only contact with the patient was when he stood outside the room for at least 2 minutes, several times a day, handing supplies to the VZV-immune nurse inside the room that was directly caring for the patient. The access to the isolation room was a standard single hinged-door that opened into the room. The non-immune nurse developed chickenpox 10 days later. Transmission of the same virus between the patient and the nurse was confirmed by viral sequencing of skin lesion samples taken from both individuals. The negative pressure difference across the doorway was measured to be only 3 Pa and it was postulated that this was readily reversed each time the door was opened to receive the supplies. This may have been a fairly typical pressure differential found in many hospital isolation units at this time, and it is clear from this incident that this was probably insufficient to maintain the containment during a door-opening motion event. Additional qualitative flow visualization studies were performed using food dye in a small-scale, water-tank model under the principle of Reynolds number equivalence, to exam the action of the hinged-door opening motion on the airflow across the doorway. These additional experiments confirmed that the hinged-door opening motion into the isolation room was likely to have caused a transient reversal of the negative pressure, allowing airborne virus to leak out and be inhaled and infect the non-immune nurse standing outside. However, the water-tank model developed for this study (with a hand-operated hinged-door) was relatively simple and crude, and this study extends this model and tests double and sliding-door options, also.

Later, Tang et al. [1], reviewed various other factors potentially involved in the aerosol transmission of infection, and also provide a simple estimate of the effect of a human body moving through the doorway, suggesting that an adult of 1.7 m height, 0.3 m width and 0.15 m depth, giving an approximate cross-sectional area of 0.51 m^2^, weighing about 76.5 kg (assuming a body density equivalent to water), walking at 1 m/s through the doorway produces an air volume flux of 255 L/s, with an attached wake of 76–230 L/s. Eames et al. [20] further refined these estimates, using a more complex, Reynolds number-equivalent, small-scale water-tank model, suggesting that the contribution from a human body wake may be as much as that produced the motion of a single hinged-door, and as much as 10% of the total room volume for a typical isolation room volume of 31 m^3^, which is similar to those described earlier by Rydock and Eian [19]. This investigation takes some aspects these earlier studies further in a qualitative manner, i.e. by investigating the impact of the moving human figure on different door designs, moving at different speeds, in a 1∶10 motorized scale water-tank model of a single isolation room.

Johnson et al. [21] simulated the effect of a moving healthcare worker using a life-sized manikin passing through a doorway made of curtains, using airborne, fluorescent beads produced by a nebulizer within the room as tracer particles. Air samplers captured escaping fluorescent beads onto filters as the manikin was moved through the curtains, both of which were moved by fine wires. Fluorescence microscopy of filters obtained from samplers placed inside and outside the room allowed a ‘particle containment efficiency to be calculated, which indicated that the manikin movement through the doorway did induce the escape of particle tracers to the outside of the room. This phenomenon is also confirmed in this study – the movement of the human figure through the doorway does induce some backflow/backwash of food dye out of the room as the figure enters.

A follow-up study from the same team [22] using similar a methodology used a real hospital isolation facility with a human volunteer to simulate the actions of a healthcare worker. They specifically stated that hinged-doors were used between the various room compartments (i.e. between the isolation room and anteroom, and anteroom and outside corridor). In this more real-life situation, they also reported that the presence of a healthcare worker moving into and out of the isolation room increased the escape of airborne particles, but also that increasing degrees of negative pressure decreased the amount of particles escaping from the isolation room. However, in this and the previously described studies, none of the teams compared and contrasted single- and double-hinged doors, and sliding doors were not investigated or discussed. This study has shown the different effects of single- and double-hinged and sliding doors on the airflow movement generated by door-opening motions alone, as well as in combination any manikin movement through the door to simulate the movement of a healthcare provider.

There are several limitations with the small-scale, water-tank model used here: no ventilation airflow has been simulated as this is a baseline study, and the airflow motion indicated by the colored food dye is only qualitative. Further experiments are required to investigate the effects of various ventilation modes, though the scaling issues for thermal buoyancy and pressure effects may make this difficult in a small-scale, water-tank model like the one used here. Computational fluid dynamical (CFD) modeling of the airflows across doorways has been performed recently [24], but this is difficult to compare directly to these experimental results as they also take into account pressure and thermal differences across the doorway, which are both equal on either side of the doorway in the baseline experiments presented here. However, one interesting finding from the CFD modeling, the phenomenon of the back-flow of potentially contaminated air when a hinged-door is opened, has been observed in our qualitative experimental findings here, and demonstrated in the case report by Tang et al. [23].

However, the conclusions from the images obtained (and in the accompanying online videos) clearly demonstrate that sliding doors induce much less airflow across the doorway than hinged-doors; single-doors cause less disturbance than double-doors (assuming that the single-doors are smaller than the double-doors, which is not always the case in some facilities); and that the movement of a single healthcare worker through the doorway in either direction induces additional airflow movement, thereby increasing the amount cross-contamination across the doorway. The case report by Tang et al. [23] suggested that door-opening motions will almost certainly reverse any low level pressure differentials (i.e. <5 Pa) across doorways, and these experiments provide a visual representation of how and why this may occur, with several combinations of door opening moving human figure parameters.

Perhaps the most important implication from this study is that whatever door design is used, there is likely to be some leakage across the doorway to a lesser or greater degree as a human figure moves through the door at a reasonable walking speed – which strongly supports the requirement for anterooms.

In the small-scale water-tank models used in these experiments, the compartment outside the isolation room, into or from which the manikin enters, can be considered as an anteroom or a corridor area. The opening of the sliding- or hinged-doors induces a variable amount of leakage (as indicated by the movement of the food dye) from the ‘dirty’ area across the doorway to the ‘clean’ area – much more so for the hinged than the sliding doors. In addition, with the moving manikin, when entering or leaving an isolation room there is clearly either a backwash or an entrained drag wake flow into this outer area, arising from the passage of a person into or out of the room, respectively. Both of these sources of contamination argue for the use of an anteroom area adjacent to the isolation room, in which the air should be completely exchanged (and filtered) before allowing an exit into the corridor. However, it is acknowledged that other more economical and space limitation factors may also influence the availability of anterooms with isolation units in individual hospitals or healthcare facilities.

It is difficult to make a direct comparison with contact transmission as to which route is more clinically significant for acquiring hospital-related infections. One recent observational study examined the number of viable bacteria found on hospital door handles of different designs in certain high traffic areas in a tertiary referral hospital in the UK. The authors found that the door handle’s location, design and mode of use were all factors that affected their degree of contamination, with the traditional lever-style handles being the most highly contaminated [25].

However, in most cases when sliding doors are installed, they are often controlled by automated detection systems that detect the approach of people and open automatically, without any touching of the door surface being required. Where a contact plate is required for opening such sliding doors (particularly those leading into dedicated isolation units), these are often ‘foot’ plates at floor level that are operated by a person’s foot pressure, or no-touch sensor plates at higher positions that just require the hand to be passed over them, without any contact with them at all. In short, the way that sliding doors are installed often preclude the need to touch these door surfaces at all, which is another advantage of these style of doors, particularly in areas where the isolation of potentially infectious (or especially vulnerable) patients is of prime clinical importance.

This study is part of an international collaboration between a small-scale modeling facility (Singapore) and a full-scale modeling facility (Finland). Further results from the large-scale modeling experiments are currently in preparation for publication. In addition, this experimental data will be used to validate the CFD modeling of these airflow patterns across the doorway under different ventilation modes, which is also being performed in both the Singapore and Finnish facilities as part of this international project.

## Supporting Information

Video S1 **Single sliding-door.** The door was programmed to open in various combinations involving the moving manikin (simulating a healthcare worker) entering/leaving the isolation room. Details of the movement parameters used for the door and manikin motions are included within the video. All experiments were performed in still water i.e. no simulated ventilation was present in these baseline experiments.
(WMV)

Video S2 **Double sliding-doors.** The doors were programmed to open in various combinations involving the moving manikin (simulating a healthcare worker) entering/leaving the isolation room. Details of the movement parameters used for the door and manikin motions are included within the video. All experiments were performed in still water i.e. no simulated ventilation was present in these baseline experiments.
(WMV)

Video S3 **Single hinged-door.** The door was programmed to open in various combinations involving the moving manikin (simulating a healthcare worker) entering/leaving the isolation room. Details of the movement parameters used for the door and manikin motions are included within the video. All experiments were performed in still water i.e. no simulated ventilation was present in these baseline experiments.
(WMV)

Video S4 **Double hinged-doors.** The doors were programmed to open in various combinations involving the moving manikin (simulating a healthcare worker) entering/leaving the isolation room. Details of the movement parameters used for the door and manikin motions are included within the video. All experiments were performed in still water i.e. no simulated ventilation was present in these baseline experiments.
(WMV)

## References

[1] Tang JW, Li Y, Eames I, Chan PK, Ridgway GL (2006) Factors involved in the aerosol transmission of infection and control of ventilation in healthcare premises. J Hosp Infect 64: 100–114. 16916564 10.1016/j.jhin.2006.05.022 PMC7114857

[2] Eames I, Tang JW, Li Y, Wilson P (2009) Airborne transmission of disease in hospitals. J R Soc Interface (Suppl 6): S697–702. 10.1098/rsif.2009.0407.focus PMC2843953 19828499

[3] Harries AD, Maher D, Nunn P (1997) Practical and affordable measures for the protection of health care workers from tuberculosis in low-income countries. Bull World Health Organ 75: 477–489. 9447782 PMC2487014

[4] Sehulster L, Chinn RY; CDC; HICPAC (2003) Guidelines for environmental infection control in health-care facilities. Recommendations of CDC and the Healthcare Infection Control Practices Advisory Committee (HICPAC). MMWR Recomm Rep 52(RR-10): 1–42. 12836624

[5] Jensen PA, Lambert LA, Iademarco MF, Ridzon R; CDC (2005) Guidelines for preventing the transmission of Mycobacterium tuberculosis in health-care settings, 2005. MMWR Recomm Rep 54(RR-17): 1–141. 16382216

[6] Humphreys H (2007) Control and prevention of healthcare-associated tuberculosis: the role of respiratory isolation and personal respiratory protection. J Hosp Infect 66: 1–5. 17350724 10.1016/j.jhin.2007.01.007

[7] Lee NE, Siriarayapon P, Tappero J, Chen KT, Shuey D, et al (2004) Infection control practices for SARS in Lao People’s Democratic Republic, Taiwan, and Thailand: experience from mobile SARS containment teams, 2003. Am J Infect Control 32: 377–383. 15525911 10.1016/j.ajic.2004.03.005 PMC7119115

[8] Fung CP, Hsieh TL, Tan KH, Loh CH, Wu JS, et al (2004) Rapid creation of a temporary isolation ward for patients with severe acute respiratory syndrome in Taiwan. Infect Control Hosp Epidemiol 25: 1026–1032. 15636288 10.1086/502339

[9] Leung TF, Ng PC, Cheng FW, Lyon DJ, So KW, et al (2004) Infection control for SARS in a tertiary paediatric centre in Hong Kong. J Hosp Infect 56: 215–222. 15003670 10.1016/j.jhin.2003.11.004 PMC7124203

[10] Liu JW, Lu SN, Chen SS, Yang KD, Lin MC, et al (2006) Epidemiologic study and containment of a nosocomial outbreak of severe acute respiratory syndrome in a medical center in Kaohsiung, Taiwan. Infect Control Hosp Epidemiol 27: 466–472. 16671027 10.1086/504501

[11] Fusco FM, Puro V, Baka A, Bannister B, Brodt HR, et al (2009) Isolation rooms for highly infectious diseases: an inventory of capabilities in European countries. J Hosp Infect 73: 15–23. 19647337 10.1016/j.jhin.2009.06.009 PMC7114849

[12] Stuart RL, Cheng AC, Marshall CL (2009) Ferguson JK; Healthcare infection control special interest group of the Australian Society for Infectious Diseases (2009) ASID (HICSIG) position statement: infection control guidelines for patients with influenza-like illnesses, including pandemic (H1N1) influenza 2009, in Australian health care facilities. Med J Aust 191: 454–458. 19835543 10.5694/j.1326-5377.2009.tb02886.x

[13] Lee S, Chowell G, Castillo-Chávez C (2010) Optimal control for pandemic influenza: the role of limited antiviral treatment and isolation. J Theor Biol 265: 136–150. 20382168 10.1016/j.jtbi.2010.04.003

[14] Harrison JP, Bukhari SZ, Harrison RA (2010) Medical response planning for pandemic flu. Health Care Manag (Frederick) 29: 11–21. 20145462 10.1097/HCM.0b013e3181cd8ab1

[15] Rice N, Streifel A, Vesley D (2001) An evaluation of hospital special-ventilation-room pressures. Infect Control Hosp Epidemiol 22: 19–23. 11198017 10.1086/501819

[16] Rydock JP (2002) A simple method for tracer containment testing of hospital isolation rooms. Appl Occup Environ Hyg 17: 486–490. 12083168 10.1080/10473220290035688

[17] Saravia SA, Raynor PC, Streifel AJ (2007) A performance assessment of airborne infection isolation rooms. Am J Infect Control 35: 324–331. 17577480 10.1016/j.ajic.2006.10.012

[18] Hayden CS 2nd, Earnest GS, Jensen PA (2007) Development of an empirical model to aid in designing airborne infection isolation rooms. J Occup Environ Hyg 4: 198–207. 17237025 10.1080/15459620601177370

[19] Rydock JP, Eian PK (2004) Containment testing of isolation rooms. J Hosp Infect 57: 228–232. 15236852 10.1016/j.jhin.2004.01.032

[20] Eames I, Shoaib D, Klettner CA, Taban V (2009) Movement of airborne contaminants in a hospital isolation room. J R Soc Interface (Suppl 6): S757–766. 10.1098/rsif.2009.0319.focus PMC2843951 19815576

[21] Johnson DL, Lynch RA, Mead KR (2009) Containment effectiveness of expedient patient isolation units. Am J Infect Control 37: 94–100. 18926600 10.1016/j.ajic.2008.05.011

[22] Adams NJ, Johnson DL, Lynch RA (2011) The effect of pressure differential and care provider movement on airborne infectious isolation room containment effectiveness. Am J Infect Control 39: 91–97. 20864218 10.1016/j.ajic.2010.05.025

[23] Tang JW, Eames I, Li Y, Taha YA, Wilson P, et al (2005) Door-opening motion can potentially lead to a transient breakdown in negative-pressure isolation conditions: the importance of vorticity and buoyancy airflows. J Hosp Infect 61: 283–286. 16253388 10.1016/j.jhin.2005.05.017 PMC7114940

[24] Beauchêne C, Laudinet N, Choukri F, Rousset JL, Benhamadouche S, et al (2011) Accumulation and transport of microbial-size particles in a pressure protected model burn unit: CFD simulations and experimental evidence. BMC Infect Dis 11: 58. 21371304 10.1186/1471-2334-11-58 PMC3056797

[25] Wojgani H, Kehsa C, Cloutman-Green E, Gray C, Gant V, et al (2012) Hospital door handle design and their contamination with bacteria: a real life observational study. Are we pulling against closed doors? PLoS One 7: e40171. 23077475 10.1371/journal.pone.0040171 PMC3471909

