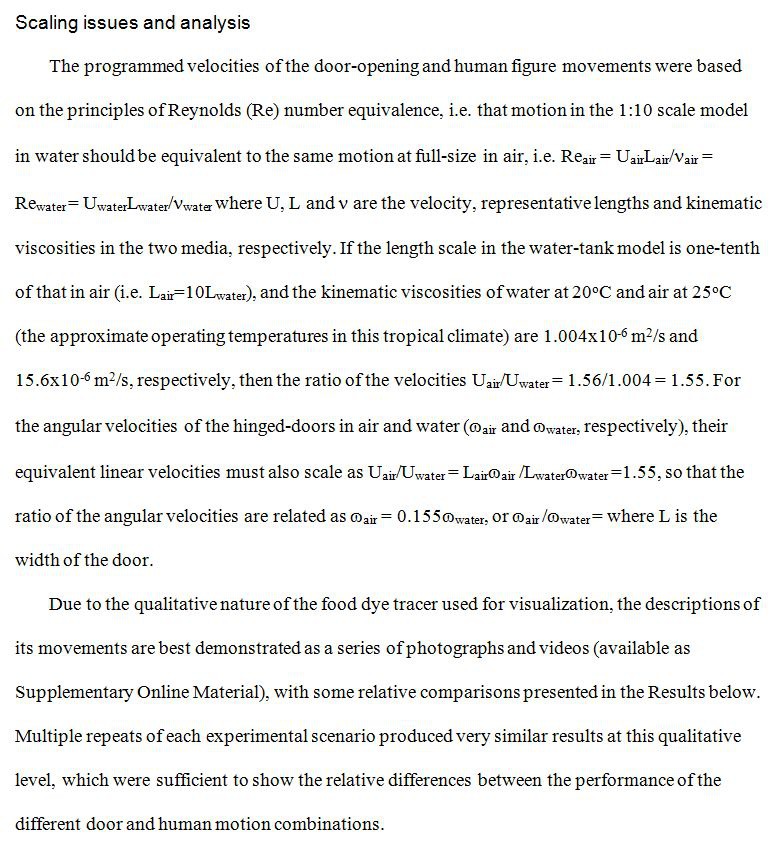# Correction: Different Types of Door-Opening Motions as Contributing Factors to Containment Failures in Hospital Isolation Rooms

**DOI:** 10.1371/annotation/c9cb4143-f96f-4529-ba37-095447a064e3

**Published:** 2014-01-06

**Authors:** Julian W. Tang, Andre Nicolle, Jovan Pantelic, Christian A. Klettner, Ruikun Su, Petri Kalliomaki, Pekka Saarinen, Hannu Koskela, Kari Reijula, Panu Mustakallio, David K. W. Cheong, Chandra Sekhar, Kwok Wai Tham

An error occurred in the "Scaling issues and analysis" sub-section of the Methods section, in which certain characters were changed to question marks. Please see the corrected paragraph here: 

**Figure:**